# Reflections on the Theoretical Prerequisites for Initial Oral Antibiotic Treatment for Paediatric Bone and Joint Infections: A Narrative Review

**DOI:** 10.3390/antibiotics15040353

**Published:** 2026-03-30

**Authors:** Pablo Rodriguez, Ahmer Khan, Giacomo De Marco, Oscar Vazquez, Andreas Tsoupras, Ardian Ramadani, Christina Steiger, Romain Dayer, Dimitri Ceroni

**Affiliations:** 1 Orthopaedics and Traumatology Unit, Surgery Department, Geneva University Hospitals, CH-1205 Geneva, Switzerland; 2 Paediatric Orthopaedics Unit, Service of Paediatric Surgery, Department of Women, Children and Adolescents, Geneva University Hospitals, CH-1205 Geneva, Switzerland

**Keywords:** paediatric, osteoarticular infection, oral antibiotic therapy, early-switch, oral-first, pharmacokinetics, pharmacodynamics, bone penetration, bioavailability

## Abstract

Paediatric osteoarticular infections (OAIs) encompass a heterogeneous group of musculoskeletal infections associated with acute septic complications, prolonged morbidity and potentially long-term sequelae. Over the past two decades, advances in microbiological diagnostics—particularly nucleic acid amplification assays—have refined the aetiological understanding of OAIs and started a new therapeutic debate regarding the most appropriate routes of antibiotic administration. Clinicians now evaluate which children can be treated safely using oral antibiotics from the outset (oral-first), which require an initial intravenous (IV) phase before a step-down to oral therapy, and which will need IV therapy all along their care pathway. Treatment debates are particularly relevant in contexts involving constrained healthcare resources and limited hospital bed availability. This narrative review summarises the essential prerequisites for prescribing oral antibiotic therapy for paediatric OAIs and proposes a pharmacokinetic/pharmacodynamic (PK/PD) framework for guiding clinical decision-making. Key considerations include: pathogen identification and resistance profiling; contemporary bacteriological epidemiology; the comparative effectiveness of IV versus oral therapy; the availability of active oral antibiotics and their penetration into bone and joint compartments; achieving adequate systemic exposure and hitting PK/PD targets after oral administration; and the clinical limitations of oral antibiotic therapy, including patient selection criteria. We argue that oral-first and early-switch strategies are best framed as structured selection processes that integrate clinical severity and source control, pathogen/minimal inhibitory concentration constraints, the feasibility of attaining PK/PD targets orally and the reliability of follow-up. No single strategy should be seen as a universal default strategy.

## 1. Introduction

Paediatric osteoarticular infections (OAIs) are serious conditions that can lead to severe septic complications, prolonged morbidity with long-term functional impairment, and disturbances to subsequent bone development. OAIs manifest themselves in a wide range of ways and affect the musculoskeletal system at different anatomical levels. In addition to the more common acute haematogenous osteomyelitis, septic arthritis and spondylodiscitis, paediatric OAIs also include atypical entities such as pyomyositis, tendon sheath infections and bursitis. Regardless of their specific presentation, prompt medical treatment—and surgical management in selected cases—is required to minimise the complications associated with delayed or inadequate care [1].

Since the early 2000s, the widespread use of nucleic acid amplification assays (NAAAs) has substantially improved microbiological diagnosis and profoundly reshaped our understanding of OAIs’ aetiology and pathophysiology, leading to major changes in diagnostic strategies and therapeutic management [2,3,4]. Despite these advances, consensus on optimal treatment strategies remains limited, particularly concerning which OAIs can be safely managed using antibiotic therapy alone and which would benefit from surgical intervention. Clinical presentations and prognoses have long been known to vary according to the causative pathogen; consequently, therapeutic strategies may diverge considerably. Infections caused by pyogenic bacteria, such as *Staphylococcus aureus* (*S. aureus*) or *Streptococcus pneumoniae* (*S. pneumoniae*), are typically associated with more severe illness, a slower clinical response and potentially worse outcomes, thereby justifying invasive diagnostic procedures and the rapid initiation of intravenous (IV) antibiotic therapy [5,6]. In contrast, OAIs caused by *Kingella kingae* (*K. kingae*) are now known to follow a milder course and may often be treated successfully with oral antibiotic therapy alone [2].

For a very long time, children with acute OAIs received prolonged IV antibiotic therapy, which, in rare cases, could then be switched to oral treatment. This classic approach has been increasingly questioned because it is associated with prolonged hospitalisation, the need for central venous access, higher complication rates and significant socioeconomic effects [7]. Consequently, several studies have proposed shortening the duration of IV therapy to just a few days—typically 2 to 7 days in uncomplicated cases—before transitioning to oral antibiotic therapy [8,9,10,11,12]. More recently, some authors have even advocated for the immediate initiation of oral antibiotic therapy, a strategy that appears attractive because of its simplicity and cost-effectiveness [13]. Before the widespread adoption of this therapy, however, oral-first strategies must be evaluated against the fundamental principles governing antibiotic efficacy.

## 2. Methodology

This narrative review aimed, therefore, to address key questions underlying this shift in therapeutic thinking, to clarify the pharmacological and experimental rationales supporting oral antibiotic therapy and to delineate the therapy’s clinical limitations. Conceptual articles examining the domain were identified through a search of the PubMed database and limited to English-language publications reporting original research. The search was conducted in PubMed, Embase and the Cochrane Library for publications between January 2000 and December 2025. The keywords retained were: paediatric, osteoarticular infection, antibiotic, treatment, early-switch and oral-first treatment. Following the recommendations of Pai et al. [14] and with the assistance of an experienced research librarian, we defined suitable Boolean combinations. Additional targeted terms—including bioavailability, pharmacokinetics, pharmacodynamics, bone penetration, and tissue concentration, as well as names of specific pathogens (*Staphylococcus aureus*, MRSA, *Kingella kingae*, *Streptococcus pyogenes*, *Streptococcus pneumoniae*) and individual antibiotics (clindamycin, trimethoprim-sulfamethoxazole, amoxicillin, cephalosporins, fluoroquinolones, linezolid)—were used to supplement the core search for the corresponding sections of the review. This strategy was supplemented by a manual search of the references found in each retrieved article to identify further potentially interesting work.

A total of 280 publications likely to be of interest were identified in the searches. Based on the reading of the abstract alone, 80 publications could immediately be excluded, their topic not corresponding to our research subject. The other 200 articles were read and studied entirely. After analysing the data and the results of these studies, only 81 were retained; they are cited in the manuscript and appear in the bibliography.

In addition, 13 references were added specifically to support the pharmacokinetic–pharmacodynamic reference table presented in Appendix A; these references are cited within that table and are listed in the bibliography.

## 3. Rationale for Oral Antibiotic Therapy in Paediatric OAIs

### 3.1. Identification of the Causative Germ and Its Antibiotic Resistance Profile

Identifying the causative organism is a cornerstone of infectious disease management: it confirms the diagnosis, enables optimisation of the antibiotic therapy and improves clinical outcomes. Determining the pathogen also enables a characterisation of its antibiotic resistance profile—a prerequisite that is particularly critical when considering oral antibiotic therapy for OAIs.

Until the early 2000s, pathogen identification relied mainly on conventional cultures obtained from blood, joint fluid or bone aspirates. Despite appropriate sampling, culture-negative results were frequent in paediatric OAIs, with 24–68% of acute haematogenous osteomyelitis and 21–70% of septic arthritis cases remaining culture-negative [15,16].

Over the last two decades, nucleic acid amplification assays (NAAAs) have markedly improved pathogen detection in paediatric OAIs. Molecular tests have enabled substantial improvements in both sensitivity and specificity, and the subsequent development of species-specific primers has made tests less prone to contamination [17,18]. Modern polymerase chain reaction techniques allow the amplification of pathogen-specific DNA sequences within hours. In addition to pathogen identification, some NAAAs can detect genes encoding antibiotic resistance mechanisms or virulence factors [19]. With continued technological progress, it is conceivable that NAAAs could increasingly complement—or replace—standard cultures for antimicrobial susceptibility testing [19].

Next-generation sequencing, the most advanced form of NAAA, is now expected to become an essential tool in clinical microbiology, shifting pathogen characterisation from phenotypic methods (staining, morphology and metabolic traits) to a genomic definition. Plasma-based, metagenomic, next-generation sequencing represents a particularly promising approach, as it may be able to detect the pathogens responsible for localised infections from circulating microbial DNA [20,21]. This concept, known as liquid biopsy, represents a revolutionary diagnostic approach, with the potential to identify the full spectrum of OAI pathogens without invasive sampling. Taken together, these advances suggest that the identification and resistance profiling of causative pathogens will increasingly become routine in paediatric OAIs. Consequently, one of the fundamental prerequisites for oral antibiotic therapy—reliable microbiological documentation—is likely to be fulfilled in almost all cases.

### 3.2. Which Pathogens Are Most Frequently Encountered in Paediatric Bone and Joint Infections?

Knowledge of the pathogens most frequently responsible for paediatric haematogenous OAIs is essential, as identification and resistance profiling directly determine whether an effective oral antibiotic therapy is available and whether its efficacy via that route has been demonstrated. In Western countries, the bacterial epidemiology of paediatric OAIs has been refined over recent years thanks to the widespread use of NAAAs.

Current data indicate that 81.9–90.7% of primary paediatric OAIs are attributable to the following pathogens, in descending order of frequency: *K. kingae*, *S. aureus* and streptococcal species [3,4,22]. *K. kingae* is currently the most frequently implicated organism, accounting for 40.9–51.0% of microbiologically confirmed OAIs, irrespective of patient age [2,3,4,22]. *S. aureus* is responsible for 33.1–36.5% of confirmed cases [3,4,22], with methicillin-resistant *S. aureus* remaining uncommon in paediatric OAI in most European countries, accounting for 3.8% of confirmed OAI and 9.4% of *S. aureus* isolates [22]. Finally, 4.5–6.6% of paediatric OAIs are caused by streptococci, mainly *Streptococcus pyogenes* (*S. pyogenes*) and *S. pneumoniae* [3,4,22].

Thus, together, these pathogens cause nearly 90% of paediatric OAIs, substantially simplifying antibiotic decision-making, including the feasibility of oral antibiotic therapy. Importantly, effective oral antibiotic options are available for these organisms. Consequently, based on current pathogen epidemiology, there are strong arguments supporting the use of oral antibiotic therapy from the outset in a substantial proportion of paediatric OAIs.

### 3.3. Is Intravenous Antibiotic Therapy More Effective than Oral Treatment for OAIs?

A medication’s optimal route of administration, including antibiotics, is the one that achieves effective systemic exposure while minimising adverse effects [23]. Drug bioavailability, therefore, is a central concept of pharmacology, defined as the extent and rate at which an active compound reaches systemic circulation after administration (whether parenteral, oral, topical or rectal) and will then be available within the organism to ensure its therapeutic effect [24,25].

Intravenous (IV) administration provides 100% bioavailability and serves as the reference standard, whereas oral administration requires gastrointestinal absorption and is subject to first-pass metabolism, resulting in lower and more variable bioavailability [26].

An oral drug’s bioavailability is influenced by multiple physiological factors, including gastric acidity, gastrointestinal motility, intestinal microflora, enzymatic activity, organ perfusion and biological barriers, as well as by drug-specific physicochemical properties. Considering these parameters is thus crucial when discussing an oral antibiotic therapy, since reduced bioavailability may influence its effectiveness [26,27].

When orally administered medications are well absorbed, providing blood levels that are virtually equivalent to those obtained with IV administration, there is no objective reason why there should be a therapeutic difference between IV and oral anti-infectives [28]. Although oral bioavailability is often used as a practical guide—with values above 90% generally considered excellent and those from 60 to 90% considered suitable for oral therapy—no strict threshold reliably predicts efficacy, which ultimately depends on values attaining the appropriate pharmacokinetic/pharmacodynamic (PK/PD) targets [29].

In addition to bioavailability, the time required for an active ingredient to reach its maximum plasma concentration (T_max_)—reflecting how rapidly an antibiotic achieves systemic exposure—is a key PK parameter, as it influences a drug concentration’s temporal profile relative to PD targets and can therefore affect the onset and magnitude of antimicrobial activity [30,31]. Finally, measures such as the minimal inhibitory concentration (MIC) or minimal bactericidal concentration (MBC) are also used to assess an antibiotic’s ability to perform these functions. For many antibiotics prescribed against paediatric OAIs—particularly β-lactams, clindamycin and macrolides—efficacy is best measured using time above MIC (fT > MIC) [32,33,34]. Clinical data indicate that maintaining free drug concentrations above the MIC for more than 80% of the dosing interval is a key determinant of therapeutic success [35]. Currently, the most common PK/PD measures used for anti-infective agents, such as time above MIC, *C*_max_/MIC, and AUC/MIC, rely on plasma concentration as the PK input value and MIC as the PD input value [36].

From a pharmacological perspective, oral administration remains the preferred route of drug delivery provided that adequate bioavailability is achievable, as it reduces the burden of care and eliminates risks inherent to prolonged intravenous access [37]. As noted above, if an oral antibiotic achieves tissue and serum concentrations comparable to those obtained intravenously, equivalent clinical efficacy and bactericidal activity can reasonably be expected [38]. The oral antibiotics currently available on the market are easier to administer, safe and achieve the desired therapeutic concentrations, thus making the oral route an ideal choice [23,39].

The time has come to debunk the erroneous beliefs and concepts prevalent among some physicians—such as that the bioavailability of IV medications is always significantly higher than that of their oral counterparts—so that patients are relieved of their symptoms earlier than if they receive a complete IV course of therapy [40,41,42]. Indeed, for most antibiotics, it has been demonstrated that physicians will essentially find the same amount of drug in the patient’s blood whether it was given intravenously or orally, and the chances of reinfection are the same.

Available PK/PD data indicate that, when adequate systemic exposure is achieved, antibiotic treatments can be just as effective when administered orally as when administered intravenously. Accordingly, antibacterial efficacy depends on the relationship between antibiotic exposure at the site of infection and pathogen susceptibility, rather than on the route of administration [32,33,34]. Thus, when high-dose oral regimens provide systemic exposure comparable to that obtained intravenously, PK/PD target attainment is expected to be similar, and no intrinsic inferiority of the oral route can be postulated [41].

### 3.4. Which Antibiotics Adequately Penetrate Bone Tissue and the Joint Space?

Achieving an antibiotic’s appropriate plasma concentration does not necessarily ensure its optimal penetration into specific tissues, such as bone or joint spaces. Moreover, antibiotic concentrations at the site of infection—particularly within bone—cannot be routinely monitored during treatment. The diffusion of antibiotics into bone tissue involves a complex interplay of biological and chemical processes. Bone’s unique structure, characterised by a dense mineral matrix and a network of living cells, constitutes a significant physical barrier to drug penetration. To reach the site of infection, antibiotics must diffuse through an extracellular matrix rich in collagen and hydroxyapatite, which can limit their diffusion and hinder the attainment of target therapeutic concentrations within bone tissue.

Thus, transport mechanisms play a significant role in an antibiotic’s penetration into bone tissue. Passive diffusion along a concentration gradient does occur, but it is likely insufficient for effective treatment given bone’s limited vascularisation. Consequently, active transport mechanisms involving specific transport proteins can contribute to an antibiotic’s cellular uptake, facilitating its passage across membranes and its accumulation within bone tissue.

The bone’s microenvironment also influences antibiotic penetration due to factors such as pH, ionic strength and the presence of binding proteins, which can modify antibiotic solubility and diffusion. The acidic environment commonly encountered in infected bone may alter an antibiotic’s ionisation state, thereby affecting its solubility and tissue diffusion. Additionally, binding proteins within bone tissue can sequester antibiotics and reduce their local availability at the infection site. Similarly, antibiotic distribution is also influenced by plasma protein binding, which determines the fraction of free, pharmacologically active medication that is available to penetrate the bone. Accordingly, antibiotics with high plasma protein binding may show reduced bone penetration, as smaller proportions of them remain unbound; this is the case for many penicillins, including cloxacillin and flucloxacillin [43,44]. Enzymatic activity within bone cells adds another layer of complexity to antibiotic penetration. Finally, bone metabolism itself may activate certain prodrugs or deactivate certain active compounds, thereby modifying antibiotic activity and efficacy. In some cases, antibiotics are designed to exploit these metabolic pathways and be converted into active forms within bone tissue. Antibiotics consequently exhibit variable abilities to penetrate and persist in bone, largely determined by their chemical structure and pharmacological properties. Numerous studies have investigated antibiotic penetration into bone and joint tissues, and these warrant a critical review [45,46,47,48].

Three classes of antibiotics are recognised for their excellent ability to penetrate and accumulate in bone: fluoroquinolones, tetracyclines and lincosamides. Tetracyclines are contraindicated for children younger than 8 years old because of their affinity for calcium ions in developing teeth, which can lead to permanent dental staining. Several other antibiotics demonstrate effective bone penetration, however, and these can represent suitable therapeutic alternatives, depending on the causative organism.

Three reviews have specifically addressed antibiotic penetration into bone and joint spaces [45,47,48]. Most of their available data were derived from studies involving antibiotic prophylaxis during orthopaedic, maxillo-facial or cardiac surgery, particularly total hip or knee arthroplasty [45,49,50,51,52,53,54].

While data on bone penetration are relatively abundant, information on antibiotic penetration into joint spaces is more limited. The overall available data indicate that most antibiotics can penetrate bone tissue and, to a similar extent, synovial fluid [45,46,47,48,49,55]. Antibiotics with documented good bone penetration include amoxicillin, piperacillin–tazobactam, flucloxacillin, cloxacillin, all four generations of cephalosporins, car-bapenems, aztreonam, aminoglycosides, fluoroquinolones, doxycycline, vancomycin, linezolid, daptomycin, clindamycin, trimethoprim–sulfamethoxazole [56], fosfomycin, rifampin, dalbavancin and oritavancin. Penicillin and metronidazole are characterised by relatively suboptimal penetration into bone tissue. Although data on antibiotic penetration into joint spaces are lacking for many agents, joint penetration is generally assumed to parallel bone penetration, with the notable exception of flucloxacillin [45,57,58]. Importantly, the available data indicate that penetration into bone and synovial tissues is primarily determined by unbound plasma concentrations rather than by the route of administration. Consequently, when an oral antibiotic therapy achieves sufficient systemic exposure, tissue diffusion is expected to be comparable to that obtained using IV administration. These considerations reinforce the biological plausibility of oral-first strategies for selected paediatric OAIs [45].

### 3.5. Availability of Effective Oral Antibiotics for the Causative Pathogen

In addition to the prerequisites for pathogen identification, antibiotic bioavailability and penetration into bone and joint tissues, the key requirement for initiating an oral antibiotic therapy for paediatric OAIs is the availability of an effective oral formulation. Some antibiotics are only available in parenteral form, which can limit oral treatment options for certain infections. Currently, however, there are several effective oral therapeutic options for each of the following pathogens responsible for approximately 90% of paediatric OAIs: *K. kingae*, *S. aureus*, *S. pyogenes* and *S. pneumoniae*.

Oral β-lactams, such as amoxicillin–clavulanic acid, can be used for methicillin-susceptible *Staphylococcus aureus* (MSSA), whereas methicillin-resistant strains (MRSA) are typically treated using agents such as trimethoprim–sulfamethoxazole, tetracyclines, clindamycin or linezolid. Oral treatments for *S. pyogenes* and *S. pneumoniae* rely primarily on amoxicillin or penicillin V. In patients with a β-lactam allergy, alternative options include oral cephalosporins (e.g., cephalexin) or macrolides (e.g., azithromycin), although macrolide efficacy may be limited by resistance. In more severe infections or when resistance is a concern, broader-spectrum agents such as amoxicillin–clavulanic acid, third-generation cephalosporins or fluoroquinolones can also be considered. Importantly, recent in vitro data have highlighted potential discrepancies between the MICs of IV and oral anti-staphylococcal β-lactams, particularly for MSSA, indicating that oral dosing regimens may require careful optimisation to achieve their PK/PD targets in more severe infections. These observations emphasise the importance of appropriate dosing strategies and cautious patient selection when oral β-lactams are used as a first-line therapy [59].

Traditionally, *K. kingae* is considered highly susceptible to most antibiotics, although β-lactamase production has occasionally been reported [60,61]. In the absence of specific guidelines for the management of invasive *K. kingae* OAIs, patients have historically been treated empirically with a variety of antimicrobial regimens [62]. In young children, empirical therapy for OAIs is therefore commonly based on penicillinase-stable β-lactams (e.g., amoxicillin–clavulanic acid) or second-generation cephalosporins with broader spectra, such as cefuroxime [2,4,17,63,64]. Taken together, these data indicate that effective oral antibiotic therapy is available for >90% of paediatric OAIs, supporting the feasibility of immediate oral treatment in selected cases.

Beyond pharmacological considerations, we must also note the practical barriers to oral therapies for children. Adherence to oral regimens may be compromised by palatability issues, particularly with high-dose liquid formulations of clindamycin or amoxicillin, and by the frequency of daily dosing and the limited availability of age-appropriate formulations for some agents [65,66]. Among young children who cannot easily swallow tablets or capsules, the feasibility of sustained high-dose oral therapy lasting several weeks should be assessed individually before committing to an oral-first or early-switch strategy.

### 3.6. Defining the Limits of Oral Antibiotic Therapy and Patient Selection

Taken together, the microbiological and pharmacological (including PK/PD) prerequisites outlined above define the essential conditions for the safe implementation of oral-first strategies. Furthermore, the success of oral antibiotic therapies for paediatric OAIs critically depends on appropriate patient and drug selection and structured clinical follow-up [13,67].

Even when the causative organism has been identified, an active oral antibiotic is available and oral dosing can achieve adequate plasma exposure, an oral-first or very early oral-switch treatment remains inappropriate for a subset of children. In practice, oral antibiotic therapy should be framed as a patient-selection strategy balancing disease severity and source-control needs against pathogen-specific constraints—particularly MIC thresholds—while ensuring reliable adherence and adequate follow-up capacity.

At the patient level, oral antibiotic therapy is commonly deferred when drug absorption or adherence is uncertain (e.g., difficulty administering medication to very young children, persistent vomiting, ileus, significant diarrhoea or malabsorption, or the inability to tolerate frequent dosing), as well as among very young infants for whom enteral absorption and PK variability may be greater and clinical deterioration more rapid. Oral antibiotic therapy should also be approached cautiously with immunocompromised patients and severely ill children (haemodynamic instability, shock, an inability to maintain hydration, or a requirement for intensive care), for whom predictable and immediate systemic exposure is a priority.

At the infection level, oral-first strategies are typically deferred when urgent source control is required (e.g., drainage of a septic joint, aspiration of abscesses or pyomyositis) or when dealing with complicated disease (multifocal osteomyelitis, a large abscess burden or epidural/paraspinal involvement). At the pathogen level, oral antibiotic therapy is inherently constrained by the PK ceiling: the maximal systemic drug exposure achievable with optimised oral dosing. When pathogen MIC values approach or exceed this threshold, established PK/PD targets cannot be reliably attained, rendering oral therapy potentially ineffective [68,69]. In cases of severe MRSA infections requiring vancomycin, or in the presence of high virulence associated with Panton–Valentine leukocidin (PVL) production, current consensus recommends dosing guided by area under the concentration-time curve (AUC), targeting an AUC/MIC ratio of 400–600, with careful therapeutic drug monitoring [70].

Across all the published observational studies, the children successfully managed using oral-first strategies shared consistent clinical characteristics, including good general condition, an absence of systemic toxicity, an uncomplicated, unifocal disease, predictable pathogen susceptibility and the ability to ensure close outpatient monitoring [13,71,72]. Oral-first and early-switch strategies also presuppose a healthcare system capable of attentive follow-up, including clinical reassessment, inflammatory marker monitoring, dose optimisation and the capacity for rapid escalation when the patient’s response is suboptimal. When these conditions cannot be fulfilled, an initial IV lead-in should remain the safer default option.

### 3.7. The Current Experience of Oral Treatments for Paediatric OAIs

Over the past two decades, the management of paediatric OAIs has progressively moved away from the historical paradigm of mandatory prolonged IV antibiotic therapy. This evolution has been driven by multiple clinical pathways demonstrating that, by carefully selecting suitable children, a rapid transition to oral antibiotic therapy can be achieved without jeopardising outcomes. Beyond early-switch strategies, some authors have explored exclusive oral-first treatments in judiciously selected low-risk patients, and they have reported favourable outcomes in structured outpatient settings [13,67]. In current practice, the key challenge is therefore not whether oral antibiotic therapy can be effective, but rather how to identify—early and safely—the subgroups of children for whom oral antibiotics are expected to be reliable and sufficient, and for whom attentive follow-up is feasible. This evolution in care is further supported by the principles of antimicrobial stewardship, which recognise route optimisation—including the timely transition from IV to oral therapy—as a key strategy for reducing unnecessary IV exposure and shortening hospital stays without compromising treatment efficacy [73,74,75]. The main features distinguishing early-switch and oral-first strategies are summarised in Appendix B.

#### 3.7.1. Early-Switch After a Short IV Lead-In: Rationale and Practical Selection

The greatest body of real-world experience in most treatment centres concerns early-switch strategies, defined as a short initial IV phase followed by an oral antibiotic therapy once a clinical improvement has been documented. Conceptually, early-switch aims to ensure immediate and predictable antimicrobial exposure during the most acute phase of infection, while reducing catheter-related complications, the length of hospital stay and healthcare costs. In prospective studies and along standardised clinical care pathways, early-switch generally corresponds to a short, initial IV treatment—typically 2 to 7 days in uncomplicated cases [8,9,10,11,12]. Transition to oral antibiotic therapy is guided by clinical improvement, a resolution of fever and a clear downward trend in C-reactive protein (CRP) levels, rather than by a fixed duration of IV treatment [9,10,11,12,67,76]. Importantly, early-switch decision-making is not purely time-driven but relies on a structured risk-stratification process. Published outpatient clinical pathways agree on a consistent framework integrating overall clinical stability, a low likelihood of complicated disease or a need for mandatory source control, and the feasibility of attentive outpatient monitoring. These principles are consistently reflected in contemporary early-switch protocols and institutional care pathways [9,10,11,12,76].

#### 3.7.2. Oral-First (Exclusive Oral Therapy from the Outset): What Do We Actually Know?

Experience with exclusively oral antibiotic therapies from the outset has primarily relied on carefully selected outpatient cohorts. In a prospective single-centre study, Alcobendas et al. reported favourable outcomes among 25 children without complications who were treated exclusively with oral antibiotics [13]. This was compared with contemporaneously hospitalised children receiving IV therapy who experienced higher rates of complications and sequelae. Importantly, the outpatient group had a markedly different baseline risk profile, including a microbiological spectrum enriched for lower-risk pathogens such as *K. kingae*, underscoring the central role of patient selection rather than the route of administration per se. More recently, using a registry-based approach, the same authors compared 64 children managed exclusively using oral therapy with 893 children initially treated intravenously: they again reported excellent outcomes in the oral-only group [67]. A key contribution of this study was the development of pragmatic, clinically applicable eligibility criteria to identify children who may be safely treated with exclusive oral antibiotics without prior intravenous therapy. The criteria delineated a low-risk disease phenotype for which clinicians could accept narrower margins for error because early clinical deterioration was less likely and outpatient reassessment was feasible. The proposed criteria included an appropriate age window (6 months to 3 years old, extendable up to 5 years in cases of *K. kingae* infection), good general condition, an absence of relevant comorbidities or sepsis, adequate oral tolerance, moderate inflammatory response (CRP < 80 mg/L; erythrocyte sedimentation rate/CRP ratio ≥ 0.67), no recent trauma, skin infection or surgery, no cervical spondylodiscitis, an absence of major local complications at presentation (e.g., large abscesses), no indication for surgery, no MRSA risk factors, and the ability to attend frequent outpatient reassessments [67]. A practical framework summarising the proposed eligibility criteria and contraindications for oral-first therapy is provided in Appendix C. A clinical decision pathway integrating these criteria is presented in Appendix D. Taken together, these observational data suggest that, when patient selection is rigorous and follow-up is structured, initial oral antibiotic therapy can achieve outcomes comparable to those of early-switch clinical pathways. However, interpretation remains limited by non-randomised study designs, selection effects and the context-specific organisation of care.

#### 3.7.3. How Categorical Should Recommendations Be Today?

Taken together, available observational outpatient and registry data suggest that oral-first therapy can achieve outcomes comparable to early-switch strategies when patient selection is rigorous and follow-up is well organised. Importantly, the favourable outcomes reported with oral-only strategies should not be interpreted as evidence of their universal applicability but rather as evidence that, in rigorously selected low-risk patients, oral antibiotic therapy can be clinically equivalent to traditional IV approaches. Nevertheless, these conclusions remain constrained by the absence of randomised comparative study designs. However, high-level comparative evidence is now emerging from trials conducted in Denmark that compared empirical initial oral versus empirical initial IV therapies and demonstrated non-inferiority in uncomplicated cases [77,78]. Accordingly, rather than positioning early-switch as a universal default treatment or oral-first as a generalised replacement, the most accurate contemporary view is that the route of administration should be considered a drug-exposure tool within a structured treatment selection framework. Early-switch remains a robust and widely applicable clinical pathway, whereas oral-first appears increasingly plausible for well-defined, low-risk subgroups—provided that attentive outpatient follow-up and a rapid escalation to IV therapy are possible if a response is not promptly favourable.

### 3.8. Future Perspectives

Future progress on decisions regarding oral-first and early-switch strategies will likely depend less on identifying a single best route of administration and more on improving risk stratification, microbiological certainty and monitoring capacity. First, randomised comparative evidence is now emerging on uncomplicated paediatric bone and joint infections, and this should be expanded to address external validity across different healthcare systems, pathogen distributions and source-control practices [77,78]. In parallel, future studies should refine and validate explicit patient eligibility criteria—such as those proposed for oral-first pathways—and assess whether these rules can be safely generalised beyond the original case contexts in which they were developed [67]. Second, future clinical pathways should integrate structured outpatient follow-up, rapid access to a reassessment and standardised triggers for treatment escalation, since oral-first strategies rely critically on the early detection of suboptimal response [67]. The outpatient-oriented models described by Alcobendas et al. illustrate how follow-up feasibility is embedded within eligibility criteria, emphasising that the organisation of care is a core determinant of safety rather than a secondary consideration [67]. Finally, an important direction for future research will be linking PK/PD modelling with clinical decision-making algorithms by integrating (i) clinical response kinetics, (ii) inflammatory marker trajectories, (iii) microbiological identification—including rapid molecular methods—and (iv) drug-specific oral exposure constraints [67]. From this perspective, oral-first and early-switch strategies should be evaluated not only in terms of relapse and complication rates but also with respect to patient-centred outcomes (comfort, quality of life), healthcare utilisation and the avoidance of IV-catheter-related harms.

In parallel, the therapeutic drug monitoring (TDM) of oral antibiotics, which is still rarely performed in routine paediatric practice, may emerge as a valuable tool for verifying that target exposures are indeed achieved after oral administration, particularly for agents with variable bioavailability or among patients with uncertain absorption [79]. Population PK models specifically developed in paediatric populations could further support personalised dose optimisation and help define oral dosing regimens that reliably attain PK/PD targets across different age groups and clinical scenarios [80,81].

## 4. Conclusions

Modern diagnostics, driven by nucleic acid amplification assays and, increasingly, by genomic approaches, have refined aetiology and made it clear that a substantial proportion of osteoarticular infections in young children are caused by pathogens with predictable susceptibility profiles. They have thereby opened the debate on how early oral antibiotic therapies can be safely initiated. From a pharmacological standpoint, the central issue is not the route of administration per se, but the ability to achieve and maintain effective antimicrobial exposure at the site of infection. When an active oral agent is available and oral intake provides adequate plasma exposure, there is no intrinsic pharmacokinetic/pharmacodynamic rationale for oral antibiotic therapy to be inferior to intravenous (IV) therapy. However, effective infection management still requires a consideration of tissue penetration, targets being dependent on minimal inhibitory concentrations and the practical determinants of exposure (absorption, adherence, dosing feasibility), as well as disease-related factors that call for urgent control of the source of infection or indicate complex infection phenotypes.

Accordingly, oral-first and early-switch strategies are best framed as candidate-selection pathways that integrate: (i) clinical stability and the absence of severe systemic illness; (ii) a low likelihood of complicated disease requiring immediate invasive management; (iii) the availability of an active oral antibiotic with feasible dosing; and (iv) the capacity for attentive follow-up and rapid escalation when response is not promptly favourable. Current clinical experience gives robust support to early-switch in appropriately selected children, while outpatient data on exclusively oral therapies from the outset are emerging, suggesting that this approach may also be safe and effective in carefully defined low-risk populations. The objective is not to replace IV therapy universally, but to use IV and oral routes as complementary tools within structured algorithms that maximise efficacy, minimise harm and reduce unnecessary hospitalisation. Future research, therefore, should prioritise harmonised selection criteria, prospective comparative studies (including non-inferiority study designs) and integrated clinical pathways that combine rapid microbiological identification with dosing informed by PK/PD and reliable outpatient monitoring.

## Figures and Tables

**Figure A1:**
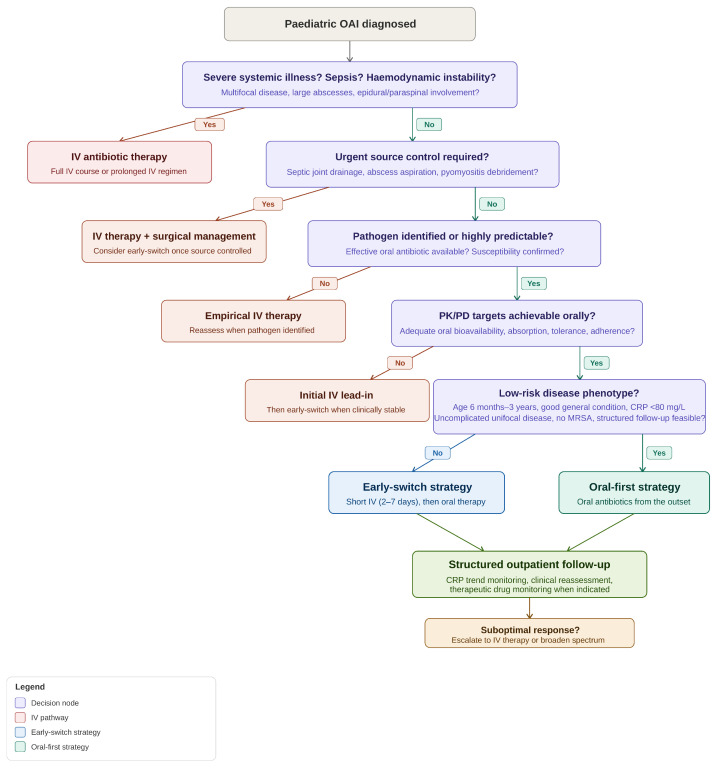
Clinical decision pathway for antibiotic route selection in paediatric OAIs.

## Data Availability

No new data were created or analysed in this study.

## Appendix A

**Table A1:** Pharmacokinetic–pharmacodynamic characteristics of antibiotics commonly used for paediatric osteoarticular infections.

Antibiotic	Key PK Properties (Oral Use)	Reported Paediatric/Human PK Data	PK/PD Target	Bone/Joint Penetration	Key Implication for Oral Therapy
Amoxicillin Amox-clav	Oral F ≈ 70% (adults); 87% (neonates). Short t½ ≈ 1–1.5 h. Non-linear (saturable) absorption at high doses.	PopPK neonates: F = 87%, 1−compartment. NAPPA study: F = 58.7%, Ka = 1.3 h^−1^ (children).	fT > MIC ≥ 40–50%. 95% PTA at 500 mg TID for MIC ≤ 0.5 mg/L.	Bone/plasma 0.10–0.20. Good synovial fluid penetration.	High-dose oral feasible for *K. kingae*, streptococci, MSSA if dosing interval ensures fT > MIC. Saturable absorption limits escalation [44,68,82,83].
Cephalexin Cefadroxil	Oral F > 90%. t½: cephalexin 1.1 h, cefadroxil 1.6 h (children). Renal elimination, no significant metabolism.	PopPK in 11 children (OAI). Cephalexin vs. cefadroxil PK in paediatric MSK.	fT > MIC ≥ 40%. Cephalexin 25 mg/kg TID: >95% PTA (MIC ≤ 4). Cefadroxil 40 mg/kg BID: >95% PTA.	Detectable in bone, abscess fluid, synovial fluid.	First-line oral step-down for MSSA OAI. Cefadroxil allows BID dosing. Frequent dosing critical for cephalexin [84,85,86].
Cefuroxime axetil	Oral prodrug; F ≈ 37–52% (variable). Food required. t½ ≈ 1.2 h.	PTA modelling, adults/children. Limited OAI-specific PK data.	fT > MIC ≥ 40–50%. PTA dependent on MIC and formulation. Suspension > tablet.	Cefuroxime IV bone/tissue data available. Oral bone PK data limited.	Variable absorption limits reliability. Less preferred than cephalexin for step-down. Main role: empirical therapy for *K. kingae* [53,87].
Clindamycin	Oral F = 87.6%, Ka = 0.967 h^−1^. CL = 15.2 L/h, V = 66.2 L (adult osteomyelitis). t½ ≈ 3 h.	PopPK, 50 adults, osteomyelitis (IV + oral). PopPK PJI (CL/F = 23 L/h). Paediatric-specific PK limited.	fT > MIC ≥ 40–50%. 600 mg TID effective up to 75 kg. C_min_ target 2 mg/L.	Bone penetration ≈ 30%. High joint/bone penetration confirmed.	Reliable oral option for MSSA/streptococci when susceptible. D-test required (inducible erm resistance). Excellent bone penetration supports oral-first [88,89,90].
TMP–SMX	TMP oral F ≈95%; SMX 85–90%. Long t½ TMP (8–11 h). Protein binding TMP ≈ 44%.	Well-established paediatric PK. Weight-based: 8–12 mg/kg/day (TMP component).	AUC_0–24_/MIC. EUCAST breakpoint *S. aureus* ≤ 2/38 mg/L. No universally accepted numeric OAI target.	Good synovial fluid penetration. Bone penetration documented in adults.	Oral option for MRSA when β-lactams unsuitable. Excellent bioavailability supports oral-first when susceptible [56].
Linezolid	Oral F ≈100%. Vd = 40–50 L; protein binding 31%. Paediatric dose: 10 mg/kg q8–12 h.	PopPK, 112 children (0–12 y). Faster clearance in children; risk of underdosing at MIC ≥ 2 mg/L.	AUC_0–24_/MIC 80–120 (adults). Dose escalation may be needed in children for MIC ≥ 2 mg/L.	Bone: serum 0.3–1.2. Mean bone concentration > 3.9 mg/L in infected tissue.	True IV–oral equivalence (F = 100%). Oral option for MRSA and VRE when tolerated. Long-term use: myelosuppression risk. TDM advisable [48,91,92,93].
Vancomycin (IV only)	No systemic oral absorption. IV only. Vd ≈ 0.4–1.0 L/kg. Renal elimination.	Well-characterised paediatric PopPK. AUC-guided dosing recommended.	AUC_0–24_/MIC 400–600 (serious MRSA). TDM mandatory.	Variable bone: serum (0.05–0.60) in adult orthopaedic studies.	IV comparator standard for severe MRSA. AUC-guided dosing not achievable orally; justifies IV route for MRSA [45,70].
Penicillin V Ampicillin	Penicillin V: F 60–73%. Amoxicillin preferred (higher F). Short t½ (0.5–1 h).	Well-established paediatric PK. Weight-based dosing standard.	fT > MIC ≥ 30–50%. TID–QID dosing required (short t½).	Bone:plasma < 0.10 for penicillin. Amoxicillin: better penetration.	Ideal for susceptible streptococci. Amoxicillin preferred over penicillin V (higher F, better bone penetration) [45,94].

**Abbreviations:** F = oral bioavailability; t½ = elimination half-life; Ka = absorption rate constant; CL = clearance; V = volume of distribution; Vd = volume of distribution; fT > MIC = fraction of dosing interval during which free drug concentration exceeds the minimal inhibitory concentration; AUC/MIC = ratio of area under the concentration–time curve to MIC; PTA = probability of target attainment; TDM = therapeutic drug monitoring; PopPK = population pharmacokinetics; OAI = osteoarticular infection; MSK = musculoskeletal; MSSA = methicillin-susceptible *S. aureus*; MRSA = methicillin-resistant *S. aureus*; VRE = vancomycin-resistant enterococci; PJI = prosthetic joint infection; BID = twice daily; TID = three times daily; QID = four times daily. Note: Only PK/PD relationships explicitly described in paediatric or high-quality human studies are reported. Data are derived from the referenced publications and should be interpreted in the context of each study’s population and methodology.

## Appendix B

**Table A2:** Comparison between early-switch and oral-first antibiotic strategies for paediatric osteoarticular infections.

Characteristic	Early-Switch Strategy	Oral-First Strategy
**Definition**	Short initial course of intravenous (IV) antibiotics, typically 2–7 days in uncomplicated cases, before a switch to oral antibiotics [8,9,10,11,12]	Exclusive oral antibiotic therapy from the outset, with no IV lead-in [13,67]
**Evidence base**	Supported by prospective cohorts, randomised trials and guideline-driven clinical practice [9,10,11,12]	Supported by observational outpatient cohorts, registry-based studies [13,67] and emerging randomised trial evidence [77,78]
**Initial care setting**	Inpatient initiation with early transition to outpatient care	Outpatient management from diagnosis
**Main rationale**	Secure early and predictable exposure while reducing IV-related complications and hospital stays	Avoid hospitalisation and IV access when oral exposure is expected to be reliable
**Clinical status at initiation**	Clinical stabilisation is achieved before switching	Good general condition at presentation; no systemic toxicity
**Inflammatory response**	Downward trend in inflammatory markers after IV initiation	Moderate inflammatory burden at onset (CRP < 80 mg/L; ESR/CRP ratio ≥ 0.67) [67]
**Disease phenotype**	Uncomplicated disease; may include cases requiring brief IV stabilisation	Strictly uncomplicated disease (single focus, no large abscesses, no epidural/paraspinal involvement, no cervical spondylodiscitis)
**Need for source control**	Performed or clearly not required before step-down to oral antibiotics	No need for urgent surgical source control (e.g., drainage of a septic joint, aspiration of abscesses or pyomyositis)
**Pathogen considerations**	Pathogen identified or predictable; IV used initially when uncertainty exists	Pathogen identified or highly predictable, with confirmed susceptibility and effective oral option (*K. kingae*, MSSA, streptococci); no MRSA risk factors
**PK/PD considerations**	IV ensures immediate attainment of PK/PD targets, followed by oral maintenance	Oral dosing must achieve PK/PD targets from the outset
**Age considerations**	Applicable across most paediatric age groups	Usually restricted to 6 months–3 years (up to 5 years for *K. kingae*); neonates and older children excluded [67]
**Host factors**	May include mild comorbidities	No significant comorbidities or immunodeficiency; no recent trauma, skin infection or surgery at the affected site
**Follow-up requirements**	Structured outpatient follow-up recommended	Mandatory attentive outpatient follow-up with rapid access to reassessments
**Risk of escalation**	Low; the early IV phase allows response assessment	Accepted but minimised by strict selection; rapid IV escalation must be feasible
**Current positioning**	Robust and widely applicable standard clinical pathway in selected children	Promising option for well-defined low-risk subgroups; not universally applicable

## Appendix C

Proposed eligibility framework for oral-first antibiotic therapy in paediatric osteoarticular infections, adapted from published criteria [67,77,78].

**Table A3:** Suggested eligibility criteria.

Criterion	Rationale
Age 6 months to 3 years (extendable up to 5 years in cases of confirmed or highly suspected *K. kingae* infection)	This age window reflects the epidemiological predominance of *K. kingae*, which is associated with predictable susceptibility and a typically milder disease course
Haemodynamically stable child in good general condition	Ensures reliable oral absorption and a low risk of rapid clinical deterioration
No underlying chronic diseases or immunodeficiency	Immunocompromised patients may require predictable IV exposure and closer monitoring
Adequate oral tolerance and reliable adherence	Oral-first strategies depend critically on sustained and adequate drug intake
Moderate inflammatory response (CRP < 80 mg/L; ESR/CRP ratio ≥ 0.67)	A lower inflammatory burden is associated with a reduced likelihood of complicated disease and supports the safety of outpatient management
No signs of sepsis or septic shock	Severe systemic illness requires immediate and predictable antimicrobial exposure via the IV route
No need for urgent surgical source control (e.g., drainage of a septic joint, aspiration of abscesses or pyomyositis)	When source control is required, an initial IV phase ensures adequate perioperative antimicrobial exposure
Uncomplicated infection (single focus, no large abscesses, no epidural or paraspinal extension)	Complicated disease phenotypes carry a higher risk of treatment failure with oral therapy alone
No cervical spondylodiscitis	Cervical location carries specific risks warranting closer inpatient monitoring
No recent trauma, skin infection or surgery at the affected site	These factors suggest possible inoculation-related infection with a potentially different microbiological profile
Pathogen identified or highly predictable, with confirmed susceptibility and an effective oral antibiotic option available (*K. kingae*, MSSA, streptococci)	Microbiological certainty is a prerequisite for reliable oral antibiotic selection
No MRSA risk factors	MRSA infections frequently require IV agents (e.g., vancomycin) with AUC-guided dosing not achievable orally
Ability to attend frequent outpatient reassessments	Oral-first strategies rely on the early detection of suboptimal response and the feasibility of rapid escalation

**Table A4:** Suggested contraindications.

Contraindication	Rationale
Age outside the validated oral-first window (<6 months or >3–5 years, depending on the pathogen)	Limited evidence and higher PK variability in neonates; different pathogen spectrum in older children
Severe systemic illness or a requirement for intensive care	Predictable and immediate systemic exposure is a priority
Multifocal osteomyelitis or extensive soft-tissue involvement	Complex disease phenotypes associated with higher failure rates
MRSA infection requiring glycopeptides and IV therapy guided by pharmacokinetics	AUC/MIC-guided vancomycin dosing cannot be achieved orally
Unknown pathogen with an unpredictable susceptibility profile, precluding reliable oral antibiotic selection	Oral-first strategies presuppose microbiological certainty or high predictability
Inability to ensure attentive clinical and biological follow-up	Without structured outpatient monitoring, early detection of treatment failure is compromised

## Appendix D

Clinical decision pathway for antibiotic route selection in paediatric osteoarticular infections (OAIs). The algorithm integrates clinical severity, source control requirements, microbiological documentation, pharmacokinetic/pharmacodynamic (PK/PD) target attainability and risk stratification to guide the choice between a full intravenous (IV) course, an IV lead-in with early switch, or an oral-first strategy. Structured outpatient follow-up is a prerequisite for both oral-first and early-switch approaches.
